# Everything counts - a method to determine viral suppression among people living with HIV using longitudinal data for the HIV care continuum - results of two large, German, multi-center real-life cohort studies over 20 years (1999–2018)

**DOI:** 10.1186/s12889-020-10088-7

**Published:** 2021-01-22

**Authors:** Daniel Schmidt, Christian Kollan, Matthias Stoll, Osamah Hamouda, Viviane Bremer, Tobias Kurth, Barbara Bartmeyer, the HIV-1 Seroconverter cohort, the ClinSurv HIV cohort

**Affiliations:** 1 Department of Infectious Disease Epidemiology, Robert Koch Institute, Berlin, Germany; 2 Institute of Public Health, Charité – Universitätsmedizin Berlin, Berlin, Germany; 3 Clinic for Rheumatology and Immunology, Infectious Diseases Unit, Medical University Hannover, Hannover, Germany

**Keywords:** Viral suppression, HIV care continuum, Treatment success, HIV cascade

## Abstract

**Background:**

The aim of this study was to develop a standardized method to reconstruct persons’ individual viral load (VL) courses to determine viral suppression and duration of viremia for the HIV care continuum in Germany using longitudinal cohort data.

**Methods:**

We analyzed data from two large, multi-center German cohort studies under the direction of the Robert Koch Institute. We included data from 1999 to 2018 of all diagnosed people and of people who initiated antiretroviral treatment (ART). We developed a model generating virtual VL values and an individual VL course corresponding to real VL measurements with a maximum distance of 180 days, considering ART status and VL dynamics. If the distance between VL measurements was > 180 days, the time between was defined as gap time. Additionally, we considered blips, which we defined as a single detectable VL < 1000 copies/ml within 180 days.

**Results:**

A total of 22,120 people (164,691 person-years, PY) after ART initiation were included in the analyses. The proportion of people with viral suppression (VL < 50 copies/ml) increased from 34% in 1999 to 93% in 2018. The proportion of people with VL < 200 copies/ml increased from 47% in 1999 to 96% in 2018. The proportion of people with viremia > 1000 copies/ml decreased from 37% in 1999 to 3% in 2018. The proportion of people with gap time fluctuated and ranged between 18 and 28%. An analysis of the first VL after gap time showed that 90% showed viral suppression, 5% VL between 50- < 1000 copies/ml and 5% VL > 1000 copies/ml.

**Conclusion:**

We provide a method for estimating viral suppression and duration of viremia using longitudinal VL data. We observed a continuous and remarkable increase of viral suppression. Furthermore, a notable proportion of those with viremia showed low-level viremia and were therefore unlikely to transmit HIV. Individual health risks and HIV drug resistance among those with low-level viremia are problematic, and viral suppression remains the goal. In 2018, 93 and 96% of people after ART initiation showed VL < 50 copies/ml and VL < 200 copies/ml, respectively. Therefore, using the threshold of VL < 200 copies/ml, Germany reached the UNAIDS 95 target of viral suppression since 2017.

**Supplementary Information:**

The online version contains supplementary material available at 10.1186/s12889-020-10088-7.

## Background

The Joint United Nations Programme on HIV/AIDS (UNAIDS) targets to accelerate the fight against HIV and to end the AIDS epidemic by 2030 aim to increase the proportion of people living with HIV (PLHIV) knowing their diagnosis, of people with diagnosed HIV infection receiving antiretroviral treatment (ART) and of people receiving ART being virally suppressed to 90% by 2020 and to 95% by 2030 [1–3].

It is estimated that, in 2018, 37.9 million people were living with HIV worldwide, and 23.3 million people were accessing ART. Globally, in 2018, 79% of PLHIV knew their status. Among people who knew their HIV status, 78% were accessing treatment. Among those people, 86% were virally suppressed. This statistic is a considerable increase in recent years compared to 2010, when only 24% of all people living with HIV were accessing treatment. New HIV infections have declined by approximately 16% since 2010 to 1.7 million new infections in 2018. Since 2010, the number of people who have died from AIDS-related illnesses worldwide has decreased by 33% to 770,000 in 2018 [4]. However, there are large differences across regions and countries regarding the HIV care continuum, with less than 50% of all people living with HIV accessing ART in Eastern Europe, central Asia, the Middle East and North Africa, and new HIV infections and AIDS-related deaths are rising in these regions [4, 5]. Additionally, for the first time since 2000, less funding was available for AIDS response in low- and middle-income countries -- almost US$ 1 billion less than in 2017 [4]. In addition, challenges with data quality, appropriate data sources and the absence of standardized definitions could hamper comparisons across countries [6].

In Germany, an increasing number of PLHIV are receiving ART [7, 8], and it is estimated that, at the end of 2018, of all PLHIV in Germany, 88% were diagnosed, and 93% of diagnosed were under ART [8]. The Robert Koch Institute (RKI) reports numbers for the German HIV care continuum to national and international stakeholders using the different available data sources. However, there is no national database containing follow-up clinical or treatment data on PLHIV. HIV surveillance, in addition to reports on diagnosed HIV/AIDS cases, requires additional surveillance tools, which are implemented with longitudinal clinical cohort studies at the RKI [9, 10]. In a former study, the RKI working group developed a method to determine the number of PLHIV receiving ART in Germany using ART prescription data and national clinical cohort data from the Clinical Surveillance of HIV Disease (ClinSurv HIV) [7]. This method, which was selected for a compendium of good HIV practices in the WHO European Region [11], has been continuously used for the second stage of the German HIV care continuum in the annual national HIV estimates of the RKI [8]. However, consistent methods for all stages of a standardized HIV continuum of care for Germany, especially for the numbers and proportions of people and their person-time with viral suppression, have not yet been published.

The main goal of ART is sustained viral suppression, which subsequently leads to several benefits. These benefits include immune recovery and decreased immune activation [12], prevention of HIV-related morbidity and mortality [13–15], reduction in non-AIDS diseases, such as cancer or cardiovascular disease [16], prevention of HIV transmission [17, 18] and avoiding the development of HIV drug resistance [19]. In Germany, effective ART evidenced by viral suppression is required by reimbursement regulations for health insurance. Hence, it is usually monitored every three months by viral load (VL) testing. Longer periods without VL controls are critical because, in cases of viral failure, immediate action would be required to avoid evolution of viral resistance or clinical progression of HIV disease.

Viral suppression is commonly defined as a VL test result below the detection limit or a certain threshold at the most recent VL test in one year [20–22]. However, such an approach does not address the dynamics of VL progression over time and could lead to biased results when the last VL is not representative of the respective year. We therefore aimed to present an alternative approach using longitudinal data, including all available VL measurements and persons’ individual ART histories.

With this study, we aimed to:

- develop a model to determine the durations and proportions of viral suppression and viremia among PLHIV to be used for the HIV care continuum;
- determine the numbers and proportions of PLHIV and of person-time with viral suppression and viremia between 1999 and 2018 using national clinical cohort data;
- compare the results of the conventional method with those of our longitudinal model; and
- evaluate the UNAIDS target of viral suppression for PLHIV in Germany.

## Methods

### HIV surveillance in Germany

National HIV/AIDS surveillance in Germany is regulated by the national Protection Against Infection Act and is based on mandatory reports of newly diagnosed cases of HIV infection and voluntary reporting of AIDS cases to the RKI, which is the federal institute of public health under the umbrella of the German Ministry of Health [23]. In addition, continuous monitoring of the course of HIV infection, including HIV treatment, is performed in HIV cohort studies at the RKI.

### Study population and data

We analyzed data from two large German cohort studies, the Clinical Surveillance of HIV Disease (ClinSurv HIV) and the HIV-1 Seroconverter cohort; both studies are under the direction of the RKI. For this analysis, cohort data between 1999 and 2018 of people with at least two VL measurements were included.

The *ClinSurv HIV cohort* is the base for a nationwide, prospective, multi-center, open, long-term observational cohort study for the clinical surveillance of HIV in Germany. Data on demographics, detailed information on the initiation, composition and discontinuation of individuals’ daily ART, laboratory parameters and clinical events are collected biannually in a standardized format. ClinSurv HIV is the largest available nationwide source of PLHIV in Germany. The study design is described in detail elsewhere [9].

The *HIV-1 Seroconverter cohort* is the basis for a nationwide, multi-center, open, long-term observational cohort study of HIV-1-positive people with a known or reliably estimated date of HIV-1 seroconversion. Sociodemographic and clinical data from each participant were collected at the time of enrollment and at yearly follow-ups. Detailed descriptions of the study methods can be found elsewhere [10, 24, 25].

### Developing a model and method to determine viral suppression and viremia

**Fig. 1:**
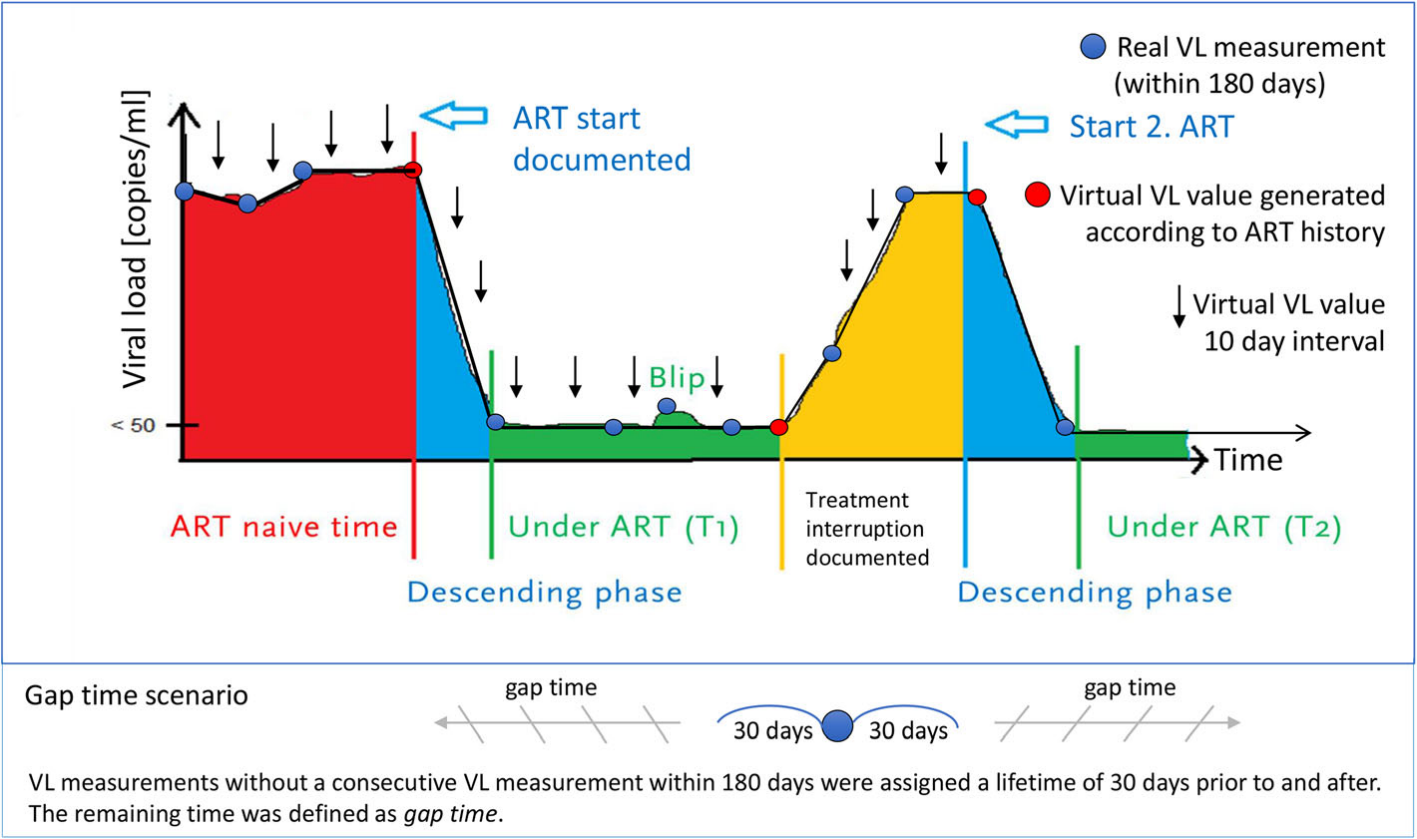
Model to determine viral suppression and viremia using longitudinal data on VL and ART history

We developed a model to reconstruct the individual VL course of people to estimate the duration and proportion of viral suppression and viremia using longitudinal data, including all available VL measurements, taking into account ART status and VL dynamics. In this model, we looked for real VL measurements with a maximum distance of 180 days. We then connected the measurements linearly and generated virtual VL values for every 10-day interval along the line. The 10-day interval was chosen because it offers sufficient accuracy with manageable data volumes. Additionally, we took into account the ART status of the people when we connected the real VL measurements. For example, if a person was coming from an ART naïve time into therapy, we did not connect the VL measurements linearly, assuming that the VL decrease started with ART initiation; therefore, a virtual VL value was generated according to the ART status, and a horizontal line was drawn from the higher VL measurement to the ART start, and then the line decreased to the lower VL measurement. Similarly, if a person’s viral load increased and an interruption were documented in the ART history, we assumed that the increase did not necessarily occur from the previous VL measurement but rather stemmed from the ART interruption (see Fig. 1). VL measurements without a consecutive VL measurement within 180 days were assigned a lifetime of 30 days prior to and after the VL measurement. The remaining time not covered by our model was defined as a longer period without VL control or so-called *gap time*. Additionally, we considered blips, which we defined as a single detectable VL < 1000 copies/ml within 180 days with a subsequent undetectable VL.

VL was a priori stratified into the following groups: VL < 50 copies/ml, VL 50- < 200 copies/ml, VL 200- < 500 copies/ml, VL 500- < 1000 copies/ml, VL 1000- < 10,000 copies/ml, VL 10,000- < 100,000 copies/ml, VL 100,000- < 1000,000 copies/ml, and VL ≥1000,000 copies/ml.

We analyzed the proportion of person-time with viral suppression and viremia over time in the total study population, indicating that, at different points in time, different people can have the same proportion of person-time and viral suppression. We also report the number and proportion of people with viral suppression and viremia, the interquartile range (IQR) and median person-time with viral suppression and viremia, and the IQR and median proportion of viral suppression and viremia to the observation time on an individual level.

Furthermore, we analyzed the time with viral suppression and viremia on an individual level, such as the proportion of people with continuous viral suppression over a period of time.

### Viral suppression and viremia from 1999 to 2018

People observed between 1999 and 2018 with at least two VLs were included. We determined viral suppression and viremia over time: (i) among all PLHIV in the cohort studies regardless of their ART status, including ART-naïve and treated person-time; and (ii) among PLHIV after ART initiation regardless of whether they were continuously under ART, including person-time with documented interruptions or gaps in treatment. Viral suppression was defined as VL < 50 copies/ml according to the German-Austrian ART guidelines [26]. When evaluating the UNAIDS target of viral suppression for PLHIV, we also report the proportion of person-time with VL < 200 copies/ml for comparability. This threshold of < 200 copies/ml for population-level monitoring is consistent with the guidelines and recommendations of several institutions, such as the US Centers for Disease Control and Prevention and the European Centre for Disease Prevention and Control [20, 27].

We determined viral suppression and viremia over time among PLHIV after ART initiation with the conventional method, using the most recent VL in each year, and we compared the results with those of our longitudinal model.

To further investigate potential misclassification using the conventional method with a single VL during one year, we determined the number and proportion of PLHIV with continuous viral suppression over a yearlong observation period on an individual level and compared it with the results of the conventional method using the most recent VL in one year.

The results on the proportion of people and person-time with viral suppression and viremia in the respective year are reported excluding gap time. The proportion of people with gap time is reported separately. Furthermore, we performed separate sensitivity analyses in the group of people with gap time to assess their VL status.

### Analysis of people with longer periods without VL control (gap time)

We report the proportion of person-time with gap time on the total observation time in the study population, the number and proportion of people with gap time, the IQR and median gap time, and the IQR and median proportion of gap time to the observation time on an individual level.

In people with gap time, the last VL measurement before and the first VL measurement after having gap time were analyzed to approximate the VL status of the people during gap time. This approach is in accordance with methods used in other studies [28, 29]. The last and first VL before and after gap time were analyzed for the recent study period from 2015 to 2018. We determined the overall proportion of people with viral suppression at the last and first VL before and after gap time. Furthermore, the congruence of the last and first VL on an individual level was determined. For the analysis of the congruence between the last and first VL before and after gap time, the proportions of people with viral suppression at both VL measurements, both VL measurements 50- < 1000 copies/ml, both VL measurements > 1000 copies/ml, the proportion of people with a VL increase (last VL < first VL) and the proportion of people with a VL decrease (last VL > first VL) were determined. Among those with detectable VL, we also report the IQR and median VL in each group.

To approximate the impact of gap time on the overall viral suppression in people who initiated ART, we calculated the resulting proportion of viral suppression after considering for viremic gap time. Therefore, we determined the proportion of viremia at the first VL measurement after gap time, determined the proportion of gap time among all people who initiated ART, then determined the resulting proportion of viremic gap time and viral suppression among all people who initiated ART.

### Analysis of antiretroviral treatment regimens over time

The ART regimens were separated into mainly used regimens according to the German-Austrian ART guidelines [26], consisting of nucleoside reverse transcriptase inhibitors (NRTIs) with either a non-nucleoside reverse transcriptase inhibitor (NNRTI), a protease inhibitor (PI), or an integrase strand transfer inhibitor (INSTI). Further ART was classified into triple-class regimens, NRTI only, NRTI-sparing regimens, attachment inhibitor (AI) containing, salvage regimens (3 drug classes and AI or fusion inhibitor (FI) or 4 drug classes), study medication, ART interruption, not fully active ART and ART gap.

We created our analytic sample in SQL Server Management Studio software, version 17.4 (Microsoft Corporation Redmond, WA, USA), and conducted the statistical analysis in Stata software, version 15.1 (StataCorp., College Station, TX, USA). Figures were created using Microsoft Excel and Microsoft Power Point 2019.

## Results

### Study population

A total of 24,569 people with at least two documented VLs from the ClinSurv HIV and the HIV-1 Seroconverter cohorts were enrolled and followed for a median of 5.9 years (IQR 2.4–11), totaling 171,990 person-years (PY). The total number of real VL measurements was 570,753, the median number of VL measurements per person was 18 (IQR 7–35), and VL monitoring occurred at a median frequency of every 91 days (IQR 64–112). With the model, 4,541,141 virtual VL values were generated. The real VL measurement and the virtual VL value occurred on the same date in 69,297 cases.

The majority of people, 88.4% (*N* = 21,716), were enrolled in the ClinSurv HIV cohort, 9.2% (*N* = 2264) were enrolled in the HIV-1 Seroconverter cohort, and 2.4% (*N* = 589) were enrolled in both cohort studies. Of the 24,569 people, 22,120 initiated ART, and 2449 were ART naïve at the end of observation. The characteristics of the study population are summarized in Table 1.

**Table 1:** Characteristics of the study population (1999-2018)

	Patients	Study population (all diagnosed PLHIV)		People who initiated ART	
		24,569	(100%)	22,120	(100%)
Observation time	Total PY	171,990		164,691	
Sex	Male	19,794	(81%)	17,794	(80%)
	Female	4775	(19%)	4326	(20%)
HIV transmission risk	Men who have sex with men (MSM)	13,006	(53%)	11,676	(53%)
	Heterosexual contacts	3227	(13%)	2954	(13%)
	High prevalence country	3200	(13%)	2958	(13%)
	People with injecting drug use	1612	(7%)	1411	(6%)
	Other	212	(1%)	203	(1%)
	Unknown	3312	(13%)	2918	(13%)
Region of origin	Germany	16,683	(68%)	15,065	(68%)
	Eastern Europe	682	(3%)	592	(3%)
	Central Europe	1129	(5%)	1021	(5%)
	Western Europe (excl. Germany)	949	(4%)	834	(4%)
	Africa	2998	(12%)	2749	(12%)
	Asia	686	(3%)	649	(3%)
	America	546	(2%)	491	(2%)
	Caribbean/Ozeania	107	(0%)	96	(0%)
	Unknown	789	(3%)	623	(3%)
Age at Enrolment (years)	Median (IQR)	37	(30-45)	37	(31-45)
Enrollment	1999–2001	3422	(14%)	3098	(14%)
	2002–2005	5454	(22%)	4834	(22%)
	2006–2009	5529	(23%)	4925	(22%)
	2010–2013	5343	(22%)	4819	(22%)
	2014–2018	4821	(20%)	4444	(20%)
Observation time (years)	Median (IQR)	5.9	(2.4-11)	6.5	(2.8-11.5)
Number of viral loads	Median (IQR)	18	(7-35)	17	(8-32)
Distance between viral loads (days)	Median (IQR)	91	(64–112)	91	(70–112)
Viral load baseline (copies/ml)	Median (IQR)	49,973	(9350–198,000)	55,544	(10,899–211,000)
CD4 cell count baseline (cells/μl)	Median (IQR)	349	(174–537)	328	(157–513)
Initiated ART	N (%)	22,120	(90%)		
ART start period	1999–2001			2416	(11%)
	2002–2005			3948	(18%)
	2006–2009			4833	(22%)
	2010–2013			5636	(25%)
	2014–2018			5287	(24%)
	not started ART			2449	(11%)
Age at ART start (years)	Median (IQR)			39	(32-46)
Time between enrollment and ART (days)	Mean (IQR)			321	(0–237)
Viral load ART start (copies/ml)	Median (IQR)			62,000	(12,300-212,604)
CD4 cell count ART start (cells/μl)	Median (IQR)			271	(133–429)
ART duration (years)	Median (IQR)			5.5	(2.3-9.9)

On an individual level, a total of 88% (21,584/24,569) achieved viral suppression at any time, and 12% (2985/24,569) never achieved viral suppression. Of all subjects, 89% (21,967/24,569) showed viremia at any time, and 82% (20,249/24,569) showed viremia with VL > 1000 copies/ml. The total median observation time was 2180 days (interquartile range (IQR) 860–4020). The median person-time with viral suppression among all people was 930 days (IQR 190–2140). The resulting individual proportion of person-time with viral suppression to the observation time had a median of 52% (IQR: 18–77). Excluding gap time, the proportion of person-time with viral suppression to the observation time had a median of 75% (IQR: 37–92). The median person-time with viremia with VL > 1000 copies/ml was 120 days (IQR 40–420). The individual proportion of person-time with viremia with VL ≥1000 copies/ml to the observation time had a median of 8% (IQR: 1.8–24). Excluding gap time, the proportion of viremia with VL ≥1000 copies/ml to the observation time had a median of 12% (IQR: 2.3–40).

### Viral suppression and viremia from 1999 to 2018

#### Among all diagnosed PLHIV

**Fig. 2:**
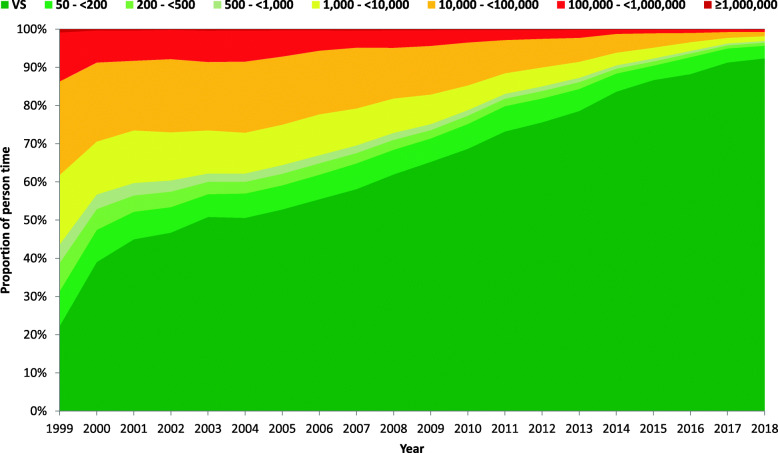
Viral load levels of all diagnosed PLHIV in the study population from 1999 to 2018

Based on the longitudinal model, the proportion of person-time with viral suppression (VL < 50 copies/ml) of the 24,569 people increased over time from 22.2% in 1999 to 92.3% in 2018. The proportion of person-time with VL < 200 copies/ml increased from 31.3% in 1999 to 95.6% in 2018. VLs of 50- < 200 copies/ml, 200- < 500 copies/ml and 500- < 1000 copies/ml were observed in 9.1, 7.4 and 4.8% of the people in 1999, respectively, and in 3.3, 0.8 and 0.4% of the people in 2018, respectively. The proportion of people with viremia > 1000 copies/ml therefore decreased from 56.4% in 1999 to 3.1% in 2018 (see Fig. 2 and Table 2 for detailed results).

**Table 2:** Development of person-time with viral suppression and viremia among all diagnosed PLHIV in the cohorts, including ART-naïve and treated person-time between 1999 and 2018 based on our longitudinal model

All diagnosed people living with HIV								
Year	Viral suppression (< 50)	50 - < 200	200 - < 500	500 - < 1000	1000 - < 10,000	10,000 - < 100,000	100,000 - < 1.000000	> 1000,000
1999	22.2	9.1	7.4	4.8	18.2	24.5	12.8	0.8
2000	39.0	8.4	5.5	3.8	13.8	20.7	8.4	0.3
2001	45.0	7.2	4.3	3.2	13.8	18.2	7.9	0.3
2002	46.7	6.7	4.1	2.9	12.6	19.2	7.6	0.2
2003	50.8	6.0	3.2	2.2	11.3	17.9	8.2	0.4
2004	50.6	6.4	3.1	2.2	10.7	18.7	8.1	0.3
2005	52.8	6.3	3.0	2.3	10.6	17.9	6.9	0.3
2006	55.5	6.4	3.0	2.1	10.7	16.7	5.3	0.3
2007	58.1	6.7	2.7	2.0	9.7	15.9	4.5	0.3
2008	62.0	6.5	2.6	1.8	9.0	13.3	4.6	0.3
2009	65.3	6.2	2.2	1.6	7.7	12.8	4.1	0.3
2010	68.7	6.5	2.2	1.5	6.5	11.2	3.2	0.2
2011	73.2	6.6	2.0	1.3	5.4	8.7	2.6	0.2
2012	75.6	6.2	2.0	1.2	5.0	7.5	2.3	0.2
2013	78.6	5.8	1.8	1.1	4.2	6.3	2.1	0.2
2014	83.6	4.8	1.3	0.9	3.3	4.9	1.1	0.1
2015 ^a^	86.7 ^a^	3.8 ^a^	1.1	0.8	2.8	3.8	0.9	0.1
2016	88.3	4.4	1.0	0.7	2.3	2.4	0.9	0.1
2017 ^b^	91.3 ^b^	3.7	0.8	0.5	1.5	1.5	0.6	0.1
2018	92.3	3.3	0.8	0.4	1.2	1.2	0.6	0.1

^a^ The UNAIDS target of viral suppression with VL < 200 copies/ml has been met for all diagnosed PLHIV in the study population in Germany since 2015

^b^ The UNAIDS target of viral suppression with VL < 50 copies/ml has been met for all diagnosed PLHIV in the study population in Germany since 2017

### People who initiated ART

A total of 22,120 people were included in the analysis with a total follow-up time of 164,691 PY, a median observation time of 6.5 years (IQR 2.8–11.5) and a median time under ART of 5.5 years (IQR 2.3–9.9). The total number of real VL measurements was 490,352, the median number of VL measurements per person was 17 (IQR 8–32), and VL monitoring occurred at a median frequency of every 91 days (IQR 70–112). With the model, 3,974,309 virtual VL values were generated. The real VL measurements and the virtual VL values occurred on the same date in 52,205 cases.

At 88.9% (*N* = 19,663), the majority were enrolled in ClinSurv HIV, 8.7% (*N* = 1936) were enrolled in the HIV-1 Seroconverter cohort, and 2.4% (*N* = 521) were enrolled in both cohort studies. The characteristics of the study population who ever initiated ART are summarized in Table 1.

On an individual level, a total of 94% (20,849/22,120) achieved viral suppression after ART initiation, and 6% (1271/22,120) never achieved viral suppression. Of all, 86% (19,076/22,120) showed viremia at any time, and 77% (17,085/22,120) showed viremia with VL > 1000 copies/ml. The total median observation time was 2010 days (IQR 850–3620). The median person-time with viral suppression among all people was 1100 days (IQR 330–2310). The resulting individual proportion of person-time with viral suppression to the observation time had a median of 66% (IQR: 36–86). Excluding gap time, the proportion of person-time with viral suppression to the observation time had a median of 88% (IQR: 63–97). The median person-time with viremia with VL > 1000 copies/ml was 40 days (IQR 10–110). The individual proportion of person-time with viremia with VL ≥1000 copies/ml to the observation time had a median of 2.3% (IQR: 0.3–9). Excluding gap time, the proportion of viremia with VL ≥1000 copies/ml to the observation time had a median of 2.9% (IQR: 0.4–13).

### Viral suppression and viremia from 1999 to 2018

#### Among PLHIV after ART initiation

**Fig. 3:**
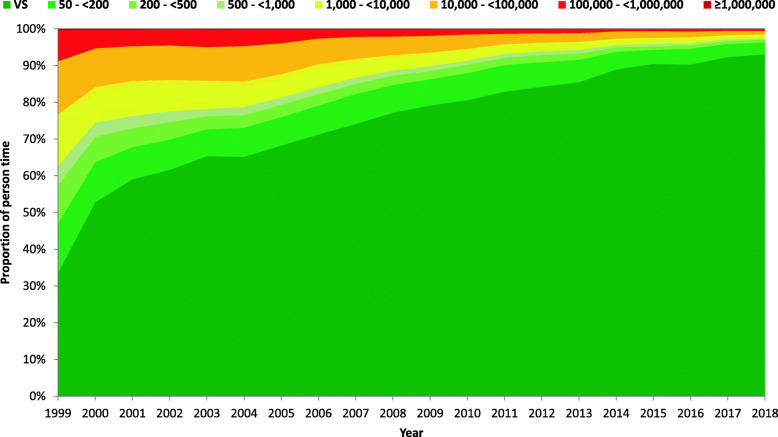
Viral load levels of people who ever initiated any ART in the study population from 1999 to 2018

Based on the longitudinal model, the proportion of person-time with viral suppression (VL < 50 copies/ml) of the 22,120 people who ever initiated any type of ART increased over time from 33.6% in 1999 to 93.0% in 2018. The proportion of person-time with VL < 200 copies/ml increased from 47.0% in 1999 to 96.3% in 2018. VLs of 50- < 200 copies/ml, 200- < 500 copies/ml and 500- < 1000 copies/ml were observed in 13.4, 10.5 and 5.2% of the people in 1999, respectively, and in 3.3, 0.8 and 0.3% of the people in 2018, respectively. The proportion of people with viremia > 1000 copies/ml therefore decreased from 37.3% in 1999 to 2.6% in 2018 (see Fig. 3 and Table 3a for detailed results).

### Viral suppression and viremia from 1999 to 2018 using the most recent VL in each year

#### Among PLHIV after ART initiation

According to a conventional definition, viral suppression, as the last step of the HIV continuum of care, is defined as the number and percentage of people receiving medical care whose most recent HIV VL is suppressed. Following this definition and considering the last VL measurement in each year, the proportion of people with viral suppression among the 22,120 people who ever initiated ART and had a documented VL value increased over time from 51.7% in 1999 to 93.3% in 2018. The proportion of people with VL < 200 copies/ml increased from 61.1% in 1999 to 96.5% in 2018. VLs of 50- < 200 copies/ml, 200- < 500 copies/ml and 500- < 1000 copies/ml were observed in 9.4, 7.4 and 3.6% of the people in 1999, respectively, and in 3.2, 0.9 and 0.3% of the people in 2018, respectively. The proportion of people with viremia > 1000 copies/ml therefore decreased from 27.9% in 1999 to 2.3% in 2018 (see Table 3b for detailed results).

**Table 3:** Development of person-time and PLHIV with viral suppression and viremia for PLHIV after ART initiation between 1999 and 2018, based on our longitudinal model (3a) and using the most recent VL in each year (3b)

People who ever initiated ART																		
Longitudinal model									Conventional method									
Year	Viral suppression (< 50)	50 - < 200	200 - < 500	500 - < 1000	1000 - < 10,000	10,000 - < 100,000	100,000 - < 1,000,000	> 1,000,000	Year	Viral suppression (< 50)	50 - < 200	200 - < 500	500 - < 1000	1000 - < 10,000	10,000 - < 100,000	100,000 - < 1,000,000	> 1,000,000	Difference in viral suppression between the methods
1999	33.6	13.4	10.5	5.2	14.0	14.5	8.4	0.4	1999	51.7	9.4	7.4	3.6	12.2	9.9	5.3	0.5	18.1
2000	52.8	10.9	6.9	3.9	9.7	10.5	5.1	0.2	2000	62.2	7.6	3.4	2.7	10.7	8.5	4.4	0.5	9.4
2001	59.1	8.7	5.2	3.3	9.5	9.5	4.4	0.3	2001	64.2	7.5	3.9	3.1	8.7	8.5	3.9	0.2	5.2
2002	61.6	8.3	4.8	2.9	8.6	9.4	4.4	0.2	2002	64.2	7.3	3.5	2.8	8.7	8.6	4.5	0.4	2.6
2003	65.3	7.3	3.5	2.0	7.7	9.1	4.7	0.2	2003	68.6	5.6	3.2	2.1	7.1	8.7	4.6	0.2	3.3
2004	65.2	7.9	3.5	2.2	7.0	9.6	4.5	0.2	2004	67.1	7.1	2.9	2.0	7.6	8.6	4.3	0.3	1.9
2005	68.2	7.7	3.4	2.0	6.3	8.4	3.8	0.1	2005	69.1	7.2	3.4	2.3	6.5	7.8	3.6	0.1	0.8
2006	71.3	7.9	3.1	1.8	6.2	7.0	2.5	0.2	2006	74.6	6.3	3.2	1.8	5.4	6.4	2.1	0.1	3.3
2007	74.1	8.1	2.8	1.7	5.1	6.1	2.0	0.1	2007	75.5	7.4	3.1	1.9	4.9	5.2	1.8	0.2	1.4
2008	77.2	7.6	2.6	1.4	4.2	5.0	2.0	0.1	2008	78.9	6.1	2.9	1.6	4.3	4.1	2.0	0.2	1.8
2009	79.1	7.2	2.2	1.3	3.7	4.6	1.7	0.1	2009	80.2	6.2	2.5	1.6	3.5	4.0	1.8	0.2	1.1
2010	80.6	7.4	2.2	1.2	3.1	4.0	1.4	0.1	2010	83.2	5.7	2.4	1.3	3.1	3.0	1.2	0.1	2.7
2011 ^a^	82.9	7.3	1.9	1.1	2.6	2.8	1.2	0.1	2011	85.1	6.0	1.7	1.1	2.4	2.3	1.3	0.1	2.2
2012	84.3	6.7	1.9	1.0	2.4	2.6	1.0	0.1	2012	85.8	5.3	2.0	1.2	2.2	2.3	1.0	0.1	1.5
2013	85.5	6.1	1.7	1.0	2.2	2.4	1.0	0.1	2013	87.7	5.1	1.8	0.9	1.8	1.8	1.0	0.1	2.2
2014	88.9	4.8	1.2	0.7	1.7	2.0	0.6	0.1	2014	90.7	3.7	1.2	0.6	1.4	1.7	0.6	0.1	1.8
2015 ^b^	90.4	3.8	1.0	0.6	1.7	1.8	0.6	0.1	2015	91.9	3.2	0.9	0.6	1.3	1.4	0.6	0.1	1.4
2016	90.3	4.3	0.9	0.6	1.6	1.6	0.6	0.1	2016	91.6	3.6	0.9	0.4	1.2	1.5	0.5	0.1	1.3
2017	92.3	3.6	0.8	0.5	1.2	1.1	0.5	0.1	2017	93.0	3.2	0.8	0.4	1.0	0.9	0.5	0.1	0.7
2018	93.0	3.3	0.8	0.3	1.1	0.9	0.5	0.1	2018	93.3	3.2	0.9	0.3	0.9	0.9	0.4	0.1	0.3

^a^ The UNAIDS target of viral suppression using the longitudinal model with VL < 200 copies/ml has been met for PLHIV after ART initiation in the study population in Germany since 2011

^b^ The UNAIDS target of viral suppression using the longitudinal model with VL < 50 copies/ml has been met for PLHIV after ART initiation in the study population in Germany since 2015

### Continuous viral suppression over one year

A total of 11,837 people with 35,995 VL measurements were eligible for the analysis of continuous viral suppression over a one-year observation period on an individual level. In total, at the individual level, 88% (10,474/11,837) had no viral failure and showed continuous viral suppression with all VLs in 2018. The median number of VLs was 3 (IQR: 2–4), and 91% (10,792/11,837) had more than one VL. Categorizing those with 1 VL or more than 1 VL measurement, 81% (848/1045) and 89% (9626/10,792) showed continuous viral suppression, respectively. In comparison, using the last VL, 93% (11,044/11,837) showed viral suppression, which is 5% higher than the proportion with continuous viral suppression on the individual level. Using all of the available VL measurements, 93% (33,619/35,995) of the VL showed viral suppression.

### Analysis of people with gap time (VL measurements > 180 days apart)

On an individual level of all 22,120 people who had ever initiated ART, 8023 (36%) had no gap time, and 14,097 (64%) had any gap time. The cumulative median gap time was 560 days (IQR: 260–1150), and the individual proportion of gap time to the observation time had a median of 27% (IQR: 12–47). The median number of gaps was 2 (IQR: 1–4), and the median gap time per gap was 223 days (IQR: 192–302).

A total of 8173 people with 15,892 VL measurements were eligible for the analysis of the last VL before and the first VL after gap time in the recent period from 2015 to 2018. Of all VL measurements, 90% (14,274/15,892) and 90% (14,293/15,892) showed viral suppression at last VL before and first VL after gap time, respectively. Furthermore, 4% (599/15,892) and 3% (531/15,892) had VL > 50- < 200 copies/ml, 1% (221/15,892) and 2% (227/15,892) had VL 200- < 1000 copies/ml, and 5% (798/15,892) and 5% (841/15,892) had VL ≥1000 copies/ml at the last VL before and the first VL after gap-time, respectively. Overall, among those with viremia, the median VL was 910 copies/ml (IQR: 104–25,700) and 1368 copies/ml (IQR: 118–31,853) for the last VL before and the first VL after gap-time, respectively.

On an individual level, of all last VLs before and first VLs after gap time, 86% were congruent with each other, with 84% showing viral suppression, 0.8% having VL 50- < 1000 copies/ml and 1.1% having VL ≥1000 copies/ml. A total of 14% were not congruent with another, with 7% having a VL increase and 7% with a VL decrease (Table 4).

**Table 4:** Congruence of last VL before and first VL after gap time, viral suppression (VS) and median VL for each group and overall between 2015-2018

Congruence	N	(%)	Last VL before gap time				First VL after gap time			
			N no VS	(%) no VS	Median	(IQR)	N no VS	(%) no VS	Median	(IQR)
Congruent VS	13,407	84.4	–		–	–	–		–	–
Congruent 50- < 1000	134	0.8	134	100	88	(66–160)	134	100	93	(66–149)
Congruent > =1000	170	1.1	170	100	33,180	(7200-83,550)	170	100	33,180	(8900–73,827)
VL decrease last VL > first VL	1051	6.6	1051	100	1162	(101–31,300)	165	16	278	(101–1820)
VL increase last VL < first VL	1130	7.1	263	23	326	(114–2555)	1130	100	1667	(135–33,824)
Total	15,892	100.0	1618	10	910	(104–25,700)	1599	10	1368	(118–31,853)

**Fig. 4:**
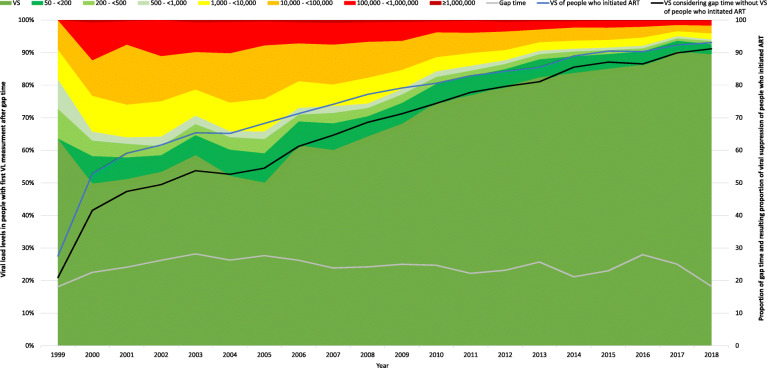
Proportion of viral suppression and viral load levels in people with gap time at their first VL measurement after gap time between 1999 and 2018. Additionally, the proportion of gap time among all people who initiated ART (grey line), the proportion of viral suppression among all people who initiated ART (blue line) and the resulting proportion of viral suppression among all people who initiated ART after considering for viremic gap time (black line) is shown

To approximate the impact of gap time on the overall viral suppression in people who initiated ART, we calculated the resulting proportion of viral suppression after considering for viremic gap time. Figure 4 shows the proportion of viral suppression and viral load levels in people with gap time at their first VL measurement after gap time between 1999 and 2018. Additionally, it shows the proportion of gap time among all people who initiated ART, the proportion of viral suppression among all people who initiated ART and the resulting proportion of viral suppression among all people who initiated ART after considering for viremic gap time. The proportion of gap time was lowest in 1999 and 2018 at 18% and highest in 2003, 2005 and 2016 at 28%, and the mean and median gap time were both 24%. The proportion of viremic gap time ranged from approximately 12% between 1999 and 2005, then decreased constantly to 2% in 2018. The resulting proportion of viral suppression among all people who initiated ART after considering for viremic gap time increased from 21% in 1999 to 90% in 2018.

### Analysis of antiretroviral treatment regimens over time

**Fig. 5:**
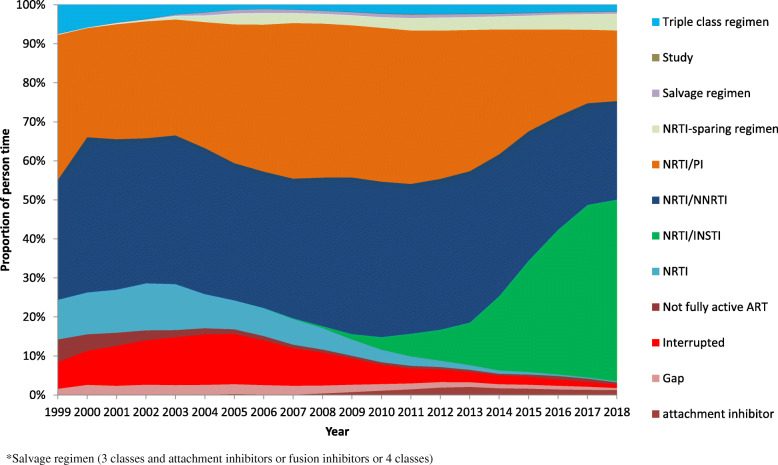
Composition of ART regimens by drug classes in the cohort studies from 1999 to 2018

The exact composition of ART regimens in the cohort studies is shown in Fig. 5 and Table S1. Overall, NRTI/NNRTI regimens with 35% were most frequently used, followed by 32% NRTI/PI regimes and 16% NRTI/INSTI regimens. The remaining 17% were divided between less common or older regimens, and 5% had treatment interruptions. The composition of ART regimens in the cohort studies changed significantly over time. Between 1999 and 2014, NRTI/PI regimens were at approximately 35%, and this proportion decreased thereafter to 18% in 2018. NRTI/NNRTI regimens ranged from approximately 35 to 40% between 1999 and 2014 and then decreased to 25% in 2018. NRTI/INSTI regimens continuously increased after their market entry in 2006, reaching 3% in 2010 and 11% in 2013 and further increasing to 47% in 2018. In 1999, a proportion of 10% was NRTI-only regimens, and this proportion decreased from 2004 to 0.4% in 2018. NRTI sparing regimens continuously increased from 0.3% in 1999 to 4% in 2018. The proportion of not fully active ART was 6% in 1999 but continuously decreased over time to only 0.5% in 2018. Interruptions were highest in 2001 to 2006 at up to 13% and then decreased continuously from 2007 onward to 1% in 2018 (see Fig. 5 and Table S1).

## Discussion

### Summary

We developed a model to reconstruct the individual viral load course of people to estimate the durations and proportions of viral suppression and viremia using longitudinal clinical cohort data, including all available VL measurements, additionally taking into account ART status and VL dynamics. The method provides a nationwide estimate and a useful method for calculating the number and proportion of PLHIV and of person-time with viral suppression for the HIV care continuum to evaluate the UNAIDS target of viral suppression for Germany. This model additionally allows for the determination and further analyses of people with longer periods without observation or missing VL control, defined as gap time. We determined the proportion of person-time and PLHIV with viral suppression and gap time between 1999 and 2018 using longitudinal national cohort data. We observed a continuous and remarkable increase in the proportion of person-time and of PLHIV being virally suppressed in both the whole study population and in PLHIV after ART initiation. The 90% UNAIDS target of viral suppression has been met in the whole study population of all diagnosed PLHIV since 2017 due to earlier and widespread use of ART and in PLHIV after ART initiation since 2015, respectively. Using the international comparable threshold of 200 copies/ml, the target was reached since 2015 and 2011, respectively. In 2018, 93% of PLHIV after ART initiation were virally suppressed with VL < 50 copies/ml, and 96% had VL < 200 copies/ml. Furthermore, we compared the results of the conventional method with those of our longitudinal method, showing potential misclassification of viral suppression when using only the last VL in a year. We observed a constant high proportion of gap time in these real-life cohort studies. We further analyzed people with gap time, aiming to approximate their viral load status, and we showed that, in recent years, only a slightly lower proportion of viral suppression was associated with gap time.

### Longitudinal model and comparison with the conventional method

Viral suppression is conventionally determined based on the most recent HIV viral load below a certain threshold, often < 200 copies/ml for comparability across studies and different settings and because this threshold was shown to be sufficient to avoid HIV transmission [17, 18, 30]. However, such a cross-sectional approach does not address the timeliness of either reaching or the time spent at each level [6] and person-time with viral suppression and viremia. Using a single VL measurement can lead to an overestimation of durable viral suppression [6, 22]. In our approach, instead of considering only the last VL measurement in a respective year, we examined the total observation time with all available VL measurements and additionally created virtual VL values taking into account ART status and VL dynamics to reconstruct persons’ individual viral load courses. We believe that this approach provides a more accurate picture of the VL status of the study population and might be especially useful when the study population and sample size are smaller and therefore less robust. When examining a large number of people cross-sectionally, it is likely that, at each point in time, a certain more or less stable proportion of people shows viral suppression. In this study, the proportion of people with viral suppression in recent years using the conventional approach with the last VL per year was ~ 2% higher than in our longitudinal model. Although this difference is small, it might be due to the large numbers of people and measurements included, which could reflect an overestimation of durable viral suppression and could be different in smaller studies or other settings. From 1999 to 2001, when the study size was smaller, the difference was ~ 11%. Furthermore, the comparison of the conventional cross-sectional approach with the analysis of continuous viral suppression in one year on an individual level showed a notable difference of 5%. Recent studies have also demonstrated that simple, cross-sectional measures of viral suppression are prone to misclassification [31]. Viral suppression is not constant once achieved, and people often transition between suppressed and unsuppressed states, even over periods as short as one year [21]. Therefore, in agreement with the results of other studies, we believe that the dynamics of VL progression are easily overlooked with a cross-sectional assessment of the last VL measure, and longitudinal measures of VL dynamics provide more granular data with implications for HIV treatment and prevention [21, 22, 31]. Additionally, with our model, it is possible to assign, quantify and further investigate longer periods without observation or VL control. For the reasons described, we believe that our method is superior when examining trends over time in longitudinal long-term cohorts with potential observation gaps and viral load changes. In addition, it should be emphasized that this advantage can be achieved on the basis of already established standards of therapy monitoring and thus with reasonable effort.

### Gap time and retention in care

In our study, we defined longer periods without viral load control of more than 180 days between VL measurements as gap time. A notable proportion of 24% gap time was observed in these real-life cohort studies. The question of whether these people are considered successfully treated or whether having viremia is a factor of uncertainty in our analysis. However, following an approach using the last VL measure for viral suppression would not consider this proportion at all. Aiming to approximate the status of the people during gap time, we analyzed the last and first VL measurements before and after gap time. On an individual level, 84% of the people came back into observation with the same VL with which they left showing viral suppression. The overall proportion of virally suppressed before and after gap time in recent years was 90%, which is only slightly (3%) less than using our longitudinal model excluding gap time. In our opinion, it is therefore very unlikely to assume that the people had high VL only during their gap time, and we believe that the VL measurements before and after gap time are good proxies. Furthermore, the median VL of the 10% with viremia before and after gap time decreased remarkably over time. Finally, we calculated the resulting proportion of viral suppression after considering for viremic gap time and showed that this would decrease the overall viral suppression by only 2% among all people who initiated ART in 2018. One reason for viral suppression or low viremia during gap time can be that people were receiving care in non-cohort centers rather than being lost to care entirely. It is important to note again that these nationwide studies are real-life observational cohorts that reflect clinical practice. People might switch doctors or leave the country or region for a certain time and then return, or it is also possible that the gaps in observation and longer periods without VL controls are in fact gaps in documentation. These might be reasons for the constant high proportion of gap time in the studies. However, VL can be very dynamic, and after ART interruption, even in selected cases with long-term viral suppression, in the absence of plasma residual viremia and low HIV-DNA or people treated in Fiebig I acute infection, viral rebound occurred rapidly at a median time of 21 or 26 days, respectively [32, 33]. Longer periods without VL control are therefore problematic. Potentially, even the quarterly reimbursed VL testing in Germany would not be sufficient to detect every single VL even if counting them as blips, and from a researcher’s perspective, we might wish to have information about the VL status of each person for every day. However, evidence has shown that quarterly VL testing is sufficient to determine treatment success, which is reflected in guidelines [26] and reimbursement regulations. Nonetheless, at least all of the available VLs should be used to determine the proportion of virally suppressed people in one year, instead of reducing the available data to only one VL per year. In our model, we use all available VLs, additionally taking into account the ART status and VL dynamics of the people to generate virtual VL values along a line, enabling us to assign a VL status at any point in observational time. We confirmed that VL testing occurred every 91 days in our cohorts, showing again that ART in Germany is performed by highly specialized practitioners in accordance with the guidelines [26]. Conversely, we also observed a constant high proportion of 24% gap time in the cohort studies, with a slightly higher likelihood of showing viremia. Retention in care is crucial for successful treatment, and we recommend maintaining engagement and retention in care and adherence to ART, accompanied, guided and monitored by regular VL testing. We also recommend further analysis among people with gap time, which we have planned. However, the achieved improvements in HIV care and treatment by highly specialized doctors are not doubted and can be seen in the composition of ART regimens over time and not least in the remarkable increase in viral suppression over time.

### Trends of viral suppression between 1999 and 2018

Between 1999 and 2018, after ART initiation, the proportions of person-time and of PLHIV with viral suppression increased from 34 to 93%. With the threshold of VL < 200 copies/ml between 1999 and 2018, the proportion of person-time and PLHIV after ART initiation increased from 47 to 96%. A remarkable increase in viral suppression has also been observed in many other studies and countries [4, 5, 34–38]. These findings are likely explained by improvements in clinical care, treatment options and ART adherence [13, 35, 38]. Although not all regimens or drugs are still being equally used, treatment options have remarkably increased since the early era after the introduction of highly active, combined ART. Not fully active ART was at 6% in 1999 but soon continuously decreased to only 0.5% in 2018. The experience of practitioners and people in using ART and the importance of adherence have improved tremendously. Resistance test-guided therapy is now the standard [26]. Much has been learned with regard to treatment interruptions, and at least since the results of the SMART study in 2006, interruptions are no longer recommended [39]. This learning can very well be seen in the proportion of interruptions in the RKI cohorts. Treatment interruptions in our study were highest at 13% in 2004 and 2005 and subsequently decreased to less than 1% in 2018. During 2015–2018 in our study population, ART interruptions still occurred in 1–2% of the people. Since ART is lifelong, we included all people who ever initiated ART even if treatment was interrupted, indicating that the proportion of viral suppression would be even higher if we restricted them to those under continuous ART. We also assessed the VL in the whole study population regardless of ART initiation, including ART-naïve people and person-time. An impressive increase was observed for all diagnosed PLHIV, which, as the results of the ClinSurv HIV and HIV-1 Seroconverter cohort showed [40], was connected with the widespread and earlier use of ART as recommended in the guidelines [26]. This achievement is a great one in terms of treatment as prevention (TasP), showing that, since early ART is common, the population of diagnosed PLHIV is not substantially contributing to HIV transmission in Germany. This fact shows that diagnosis is key to prevention. With regard to the whole HIV care continuum, we know that the potential for improvement is mostly seen at this first stage of the HIV care continuum -- the only stage for which Germany has not yet met the UNAIDS target of 90% [8]. Therefore, tailored HIV testing campaigns and enhanced access to HIV testing, including self-testing, should be further strengthened. For PLHIV after ART initiation, we recommend avoiding treatment interruptions and emphasizing adherence to ART.

### Evaluation of the UNAIDS 90 target of viral suppression

The UNAIDS target of 90% viral suppression has been met among PLHIV who ever initiated ART since 2015 in these nationwide German cohort studies of PLHIV. The international comparable threshold of VL < 200 copies/ml has been met among PLHIV who ever initiated ART since 2011. In 2018, 93% of PLHIV after ART initiation were virally suppressed with VL < 50 copies/ml, and 96% had VL < 200 copies/ml. Therefore, when using the threshold of VL < 200 copies/ml, Germany reached the UNAIDS 95 target of viral suppression since 2017. On a population basis in light of the HIV transmission risk, studies have suggested that a VL up to 400 copies/ml might still be uncritical [41]. A notable proportion of our study population with viremia showed low-level viremia < 1000 copies/ml with a likely low risk of transmitting HIV [30]. However, individual health risks [42], the development of HIV drug resistance [43–45] and increased risk of viral rebound [46–48] among people with low-level viremia are problematic, and viral suppression remains the goal [49]. Therefore, further analysis of people with viral failure is essential.

## Limitations

Assessing stages of the HIV continuum of care using cohorts can introduce bias since they might not be representative of all diagnosed PLHIV in a country. To estimate representativeness, we compared the demographic characteristics of our study population with all PLHIV in Germany and found them to be similar. The study population represents more than 20% of all PLHIV in Germany. ClinSurv HIV is the largest nationwide long-term cohort of HIV-positive people and the least biased source available. In a study by Gourlay et al., the authors also used country-specific cohort data to derive stages of the HIV care continuum in European Union countries [6]. Germany delivered data from ClinSurv HIV for this study and was assumed to be fairly representative of HIV people in care [6, 50, 51]. People outside of medical care, e.g., without health insurance, are not represented in ClinSurv. The HIV-1 Seroconverter cohort is assumed to be representative of men who have sex with men (MSM) in Germany [52] and therefore covers one specific group within the population of PLHIV. However, MSM is the largest group and is mainly affected by HIV; furthermore, ClinSurv HIV accounts for more than 90% of the study population in our analytic sample. In this respect, we believe that the representativeness of ClinSurv HIV applies, and following the results of Gourlay et al., our sample is fairly representative of HIV people in care in Germany. However, we cannot exclude that these studies are not representative.

As discussed, gap time is a factor of uncertainty, and although we believe that our sensitivity analysis of the last and first VL before and after gap time is a reasonable approximation, viral suppression was slightly lower, indicating that further analyses of people with gap time would be useful. Furthermore, in real-life studies, misclassification, loss to follow-up, lab-related issues and gaps in documentation can occur and influence gap time.

## Conclusions

This report describes a model to estimating the number and proportion of PLHIV and person-time with viral suppression. The study provides a possible approach for estimating the number of people receiving continuously specialized HIV medical care in Germany and those with gaps in observation or VL control. With this study, we provide a nationwide estimate and a useful tool for calculating the number and proportion of PLHIV and of person-time with viral suppression and with gap time as well as trends of viral suppression and gap time between 1999 and 2018 in Germany. We observed an increase in the proportion of person-time and of diagnosed PLHIV with viral suppression. The UNAIDS 90 target of viral suppression has been met in these nationwide German cohort studies since 2015 and, when using the international comparable threshold of < 200 copies/ml, since 2011. In 2018, 93% of PLHIV after ART initiation were virally suppressed with VL < 50 copies/ml, and 96% had VL < 200 copies/ml. Germany reached the UNAIDS 95 target of viral suppression since 2017 when using the threshold of VL < 200 copies/ml. Our results suggest that the population of diagnosed PLHIV is not substantially contributing to HIV transmission in Germany. Continuous efforts toward tailored HIV testing campaigns and enhanced access to HIV testing, including self-testing, are recommended.

We also recommend regular VL testing and engagement and retention in care as well as adherence to continuous ART. Further analysis of people with viral failure is essential to understand and determine risk factors for viral failure in times of highly effective and mostly successful ART.

This approach and model to reconstruct persons’ individual viral load course can be useful for estimating the number and proportion of PLHIV with viral suppression in other countries, provided that the required resources are available. The described methodology could be used and adapted for different investigations or parameters in the future.

## Supplementary Information

**Additional file 1.**

## Data Availability

The datasets generated and/or analyzed during the current study are not publicly available due to data protection and confidentiality but are available from the corresponding author on reasonable request.

## Abbreviations

AI: Attachment inhibitor
ART: Antiretroviral treatment
ClinSurv HIV: Clinical Surveillance of HIV Disease
FI: Fusion inhibitor
INSTI: Integrase strand transfer inhibitor
IQR: Interquartile range
MSM: Men who have sex with men
NRTI: Nucleoside reverse transcriptase inhibitor
NNRTI: Non-nucleoside reverse transcriptase inhibitor
PI: Protease inhibitor
PLHIV: People living with HIV (PLHIV)
PY: Person-years
RKI: Robert Koch Institute
UNAIDS: Joint United Nations Programme on HIV/AIDS
VL: Viral load
