# Supplementary material for: Everything counts - a method to determine viral suppression among people living with HIV using longitudinal data for the HIV care continuum - results of two large, German, multi-center real-life cohort studies over 20 years (1999–2018)
Source: BMC Public Health. 2021 Jan 22;21:200. doi: 10.1186/s12889-020-10088-7 (PMC7825204; doi:10.1186/s12889-020-10088-7)
Supplement: Supplementary file 1 — Additional file 1. [file 12889_2020_10088_MOESM1_ESM.docx]

Table S1. Composition of ART regimens by drug classes in the cohort studies from 1999 to 2018

| **Year** | **NRTI**  **/NNRTI** | **NRTI**  **/PI** | **NRTI**  **/INSTI** | **ART**  **interruption** | **NRTI** | **NRTI-sparing  regimen** | **Triple class  regimen** | **ART**  **gap** | **Attachment  inhibitors** | **Not fully  active ART** | **Salvage regimen** | **Study** |
| --- | --- | --- | --- | --- | --- | --- | --- | --- | --- | --- | --- | --- |
| 1999 | 30.7% | 37.2% | 0.0% | 6.8% | 10.2% | 0.3% | 7.3% | 1.6% | 0.0% | 5.8% | 0.0% | 0.1% |
| 2000 | 39.8% | 28.0% | 0.0% | 8.7% | 10.8% | 0.2% | 5.7% | 2.6% | 0.0% | 4.2% | 0.0% | 0.0% |
| 2001 | 38.5% | 29.4% | 0.0% | 10.2% | 11.0% | 0.4% | 4.5% | 2.4% | 0.0% | 3.4% | 0.1% | 0.1% |
| 2002 | 37.2% | 30.0% | 0.0% | 11.4% | 12.0% | 0.5% | 3.6% | 2.7% | 0.0% | 2.6% | 0.1% | 0.1% |
| 2003 | 38.1% | 29.7% | 0.0% | 12.3% | 11.8% | 1.0% | 2.4% | 2.5% | 0.0% | 1.8% | 0.3% | 0.0% |
| 2004 | 37.4% | 32.2% | 0.0% | 12.9% | 8.8% | 1.8% | 1.9% | 2.6% | 0.0% | 1.6% | 0.8% | 0.1% |
| 2005 | 35.1% | 35.5% | 0.0% | 12.9% | 7.4% | 2.9% | 1.2% | 2.5% | 0.2% | 1.2% | 0.9% | 0.0% |
| 2006 | 34.9% | 37.7% | 0.1% | 11.5% | 7.1% | 3.0% | 1.0% | 2.4% | 0.1% | 1.1% | 1.0% | 0.0% |
| 2007 | 35.8% | 39.9% | 0.2% | 9.6% | 6.6% | 2.6% | 1.1% | 2.3% | 0.1% | 0.9% | 0.9% | 0.1% |
| 2008 | 38.1% | 39.4% | 0.5% | 8.4% | 5.5% | 2.5% | 1.4% | 2.0% | 0.4% | 0.8% | 0.7% | 0.1% |
| 2009 | 40.2% | 39.0% | 1.3% | 6.8% | 4.2% | 2.6% | 1.7% | 1.9% | 0.8% | 0.7% | 0.7% | 0.2% |
| 2010 | 39.8% | 39.4% | 3.2% | 5.0% | 3.3% | 2.8% | 2.1% | 1.7% | 1.1% | 0.6% | 0.8% | 0.2% |
| 2011 | 38.4% | 39.3% | 5.8% | 3.8% | 2.5% | 3.2% | 2.3% | 1.5% | 1.5% | 0.7% | 0.8% | 0.3% |
| 2012 | 38.7% | 38.0% | 7.9% | 3.2% | 1.7% | 3.4% | 2.3% | 1.4% | 1.9% | 0.6% | 0.7% | 0.2% |
| 2013 | 38.8% | 36.2% | 10.8% | 2.7% | 1.3% | 3.3% | 2.2% | 1.2% | 2.1% | 0.5% | 0.7% | 0.2% |
| 2014 | 36.3% | 32.0% | 19.0% | 2.2% | 0.9% | 3.3% | 2.1% | 1.1% | 1.7% | 0.5% | 0.6% | 0.2% |
| 2015 | 33.1% | 26.1% | 28.5% | 2.0% | 0.7% | 3.6% | 2.0% | 1.0% | 1.7% | 0.6% | 0.6% | 0.2% |
| 2016 | 29.1% | 22.2% | 37.0% | 1.6% | 0.5% | 3.9% | 1.8% | 1.0% | 1.4% | 0.9% | 0.5% | 0.2% |
| 2017 | 26.0% | 18.9% | 44.2% | 1.2% | 0.4% | 4.0% | 1.7% | 0.8% | 1.3% | 0.9% | 0.4% | 0.2% |
| 2018 | 25.2% | 18.1% | 46.5% | 0.9% | 0.4% | 4.3% | 1.6% | 0.5% | 1.3% | 0.5% | 0.5% | 0.2% |
| Total | 34.9% | 31.8% | 15.9% | 4.8% | 3.1% | 3.1% | 2.0% | 1.5% | 1.2% | 0.9% | 0.6% | 0.2% |

Salvage regimen (3 classes and attachment inhibitors or fusion inhibitors or 4 classes)
